# Low‐pathogenicity influenza viruses replicate differently in laughing gulls and mallards

**DOI:** 10.1111/irv.12878

**Published:** 2021-06-10

**Authors:** Miria F. Criado, Kira A. Moresco, David E. Stallknecht, David E. Swayne

**Affiliations:** ^1^ Southeast Poultry Research laboratory United States National Poultry Research Center Agricultural Research Service U.S. Department of Agriculture Athens GA USA; ^2^ Southeastern Cooperative Wildlife Disease Study Department of Population Health College of Veterinary Medicine University of Georgia Athens GA USA

**Keywords:** avian influenza, laughing gulls, low‐pathogenicity avian influenza, mallard, pathogenesis, pathogenicity, wild birds

## Abstract

Wild aquatic birds are natural reservoirs of low‐pathogenicity avian influenza viruses (LPAIVs). Laughing gulls inoculated with four gull‐origin LPAIVs (H7N3, H6N4, H3N8, and H2N3) had a predominate respiratory infection. By contrast, mallards inoculated with two mallard‐origin LPAIVs (H5N6 and H4N8) became infected and had similar virus titers in oropharyngeal (OP) and cloacal (CL) swabs. The trend toward predominate OP shedding in gulls suggest a greater role of direct bird transmission in maintenance, whereas mallards shedding suggests importance of fecal‐oral transmission through water contamination. Additional infectivity and pathogenesis studies are needed to confirm this replication difference for LPAI viruses in gulls.

## 1 INTRODUCTION

Low‐pathogenicity avian influenza viruses (LPAIVs) have been identified in many bird species, but primarily from the orders Anseriformes (ducks, geese, and swans) and Charadriiformes (gulls, terns, and shorebirds).^1^ Mallards (*Anas platyrhynchos*) and other dabbling duck species are important LPAIV hosts, and transmission between ducks occurs through the fecal‐oral route involving contaminated water.^1^, ^2^ However, gulls also are susceptible and can contribute to geographic spread, reassortment, and the evolution of AIVs.^3^, ^4^, ^5^ Surveillance data indicate that the prevalence of AIV and subtype diversity vary significantly between different genera and species. All HA subtypes have but detected in ducks and gulls, but the H3 and H4 subtypes predominate in ducks, and H13 and H16 in gulls.^1^, ^3^, ^4^, ^5^

In this study, our goals were to understand AIV infectivity and pathogenesis in gulls, through clinical assessment, viral shedding patterns, and seroconversion, to related findings to potential mechanisms of transmission and ecological maintenance. Such experimental studies with gulls have been previously conducted on H13 and H16 viruses but not on less prevalent LPAIV subtypes. We performed experiments using the laughing gulls (*Leucophaeus atricilla*) and mallards challenged with North American LPAIVs that were originally isolated from either gulls or mallards, respectively.

## 2 METHODS

North American LPAIVs used in experiments are listed in Table 1; they were propagated in specific pathogen‐free (SPF) 9‐ to 11‐day‐old embryonating chicken eggs (ECE) following standard procedures.^6^ Low‐passage virus stocks were used for challenge.

**TABLE 1:** Avian influenza viruses and back titers used in the experimental challenge of laughing gulls and mallards

Avian species	Number of birds	Experimental Group Abbreviation	Inoculated LPAI virus strain	GenBank accession numbers (Hemagglutinin gene)	Back Titers of inoculum (EID_50_/0.1ml)
Laughing gulls	3	LG/H7N3	A/laughing gull/New York/AI00‐2455/2000 ‐H7N3*	CY144292.1	10^6.6^
	3	LG/H6N4	A/laughing gull/New York/AI00‐470/2000 ‐H6N4*	CY144162.1	10^5.5^
	3	LG/H3N8	A/laughing gull/New Jersey/768/2005 H3N8*	GU186466.1	10^5.5^
	3	HG/H2N3	A/herring gull/New York/AI00‐532/2000 ‐H2N3*	CY144178.1	10^5.6^
	2	SHAM	NA	NA	NA
Mallards	3	M/H5N6	A/mallard/Wisconsin/34/1975 ‐H5N6^#^	U79451.1	10^5.5^
	3	M/H4N8	A/mallard/Ohio/338/1986 ‐H4N8^#^	DQ021863.1	10^5.7^
	2	SHAM	NA	NA

Note

LPAI virus isolates were provided by Southeast Cooperative Wildlife Disease Study, Athens, GA (*), and Department of Veterinary Preventive Medicine, Columbus, OH (#).

Abbreviation: NA, not applicable.

Laughing gulls (7–10‐days‐of‐age) were obtained under federal permit and reared for 12 weeks in captivity until challenged. Ten‐ to 16‐week‐old mallards were purchased from a commercial hatchery (Chenoa Waterfowl). For challenge (Table 1), birds were grouped and housed in negative pressure high‐efficiency particle air (HEPA) ventilated cabinets with ad libitum access to feed and water.

Laughing gulls and mallards were divided in groups and inoculated with respective gull‐ and mallard‐origin LPAIV (Tables 1 and 2) via the choanal cleft, which provides exposure to upper respiratory tract and drainage into oral cavity for swallowing and exposure to gastrointestinal tract, with approximately 10^6^ mean embryo infectious doses (EID_50_) in 0.1 ml per bird. Back titers were reported in Table 1. Oropharyngeal (OP) and cloacal (CL) swabs were collected on 1, 2, 3, 4, 7, and 10 days of post‐inoculation (dpi) and placed in Becton‐Dickinson BBL brain heart infusion (BHI) medium with 2× concentration of antibiotics (10,000 U/ml Penicillin G, 10,000 μg/ml Streptomycin, 25 μg/ml Amphotericin B) (HyCone Laboratories, Inc). Samples were stored at −8°C until tested. Blood was collected pre‐(0‐day) and post‐inoculation (10 days) to assess serum antibody responses. Birds were observed daily for clinical signs and euthanized at 10 dpi following approved protocols. These studies were reviewed and approved by the USNPRC Institutional Animal Care and Use Committee (IACUC) and conducted with appropriate biocontainment and biosafety measures.

**TABLE 2:** Summary of OP and CL virus shedding and anti‐influenza antibodies post‐LPAI virus inoculation in laughing gulls and mallards

Avian Species	Experimental Group Abbreviation	Virus Shedding					AIV antibody+ /Total			
		Virus Detection/Total of birds	OP Swab		CL Swab		Pre‐ challenge		Post‐challenge	
			Mean Peak Titer^a^ (dpi)	Duration (day)	Mean Peak Titer^a^ (dpi)	Duration (day)	HI (mean titer^b^)	bELISA	HI (mean titer^b^)	bELISA
Laughing Gulls	LG/H7N3	3/3	10^4.5^ (1 dpi)	1–10	10^1.6^ (4 dpi)	2–4	0/3 (2^2.0^)	0/3	3/3 (2^6.3^)	3/3
	LG/H6N4	3/3	10^6.3^ (1 dpi)	1–4	10^2.9^ (2 dpi)	2–3	0/3 (2^2.0^)	0/3	3/3 (2^9.6^)	2/3
	LG/H3N8	3/3	10^1.5^ (1 dpi)	1–2	–	–	0/3 (2^2.0^)	0/3	0/3 (2^2.0^)	1/3
	HG/H2N3	1/3	10^1.6^ (1 dpi)	1–2	–	–	0/3 (2^2.0^)	0/3	1/3 (2^3.0^)	1/3
	SHAM	0/2	–	–	–	–	0/2 (2^2.0^)	0/2	0/2 (2^2.0^)	0/2
Mallards	M/H5N6	3/3	10^4.1^ (4 dpi)	1–10	10^4.7^ (4 dpi)	2–10	0/3 (2^2.0^)	0/3	3/3 (2^6.3^)	3/3
	M/H4N8	3/3	10^2.4^ (2 dpi)	1–10	10^2.4^ (2 dpi)	1–10	0/3 (2^2.0^)	0/3	2/3 (2^3.7^)	2/3
	SHAM	0/2	–	–	–	–	0/2 (2^2.0^)	0/2	0/2 (2^2.0^)	0/2

–, no virus detected

^a^ Mean Peak Titer are report as EID_50_/ml.

^b^ HI titers expressed as geometric mean titers (GMT‐log2). The HI results were determined using challenge virus as antigen. Samples with titers below 3 log_2_ GMT were considered negative, and then assigned as 2 log_2_ GMT for statistical purpose.

OP and CL swabs were processed to determine viral shedding titers by quantitative real‐time PCR (RRT‐PCR). Briefly, the RNA was extracted using MagMAXTM‐96 AI/ND Viral RNA Isolation Kit^®^ (ThermoFisher Scientific) following the manufacturer's instruction. Further, RRT‐PCR that targets the matrix gene of avian influenza was performed with the AgPath‐ID OneStep RT‐PCR kit (ThermoFisher Scientific) using 7500 FAST Real‐time PCR System (Applied Biosystems), as previously described.^7^ Virus quantity was established with a standard curve from RNA extracted from 10‐fold dilutions of the challenge virus in duplicate.

Serum was tested for anti‐AIV antibodies using hemagglutination inhibition (HI) assay and blocking enzyme‐linked immunosorbent assay (bELISA). The homologous antigens were prepared as previously described^6^ and the HI assay performed following standard procedures.^6^ Titers were calculated as the reciprocal of the last HI positive serum dilution and were converted to log_2_. Titers were expressed as geometric mean titers (GMT‐log_2_). Samples were considered positive for the presence of AI antibodies with titers ≥3 log_2_ GMT. The blocking‐ ELISA, the Avian Influenza Virus Antibody Test Kit, MultiS‐Screen Ab (IDEXX, Westbrook, Maine) was used in duplicate following the manufacture's instruction.

Statistical analyses were performed using Prism 8 (GraphPad Software).

## 3 RESULTS

The sham controls were not infected based on lack of pre‐ and post‐challenge AIV antibodies and negative virus detected in OP and CL swabs after challenge. Clinical signs or mortality were not observed in any inoculated laughing gulls and mallards.

**FIGURE 1:**
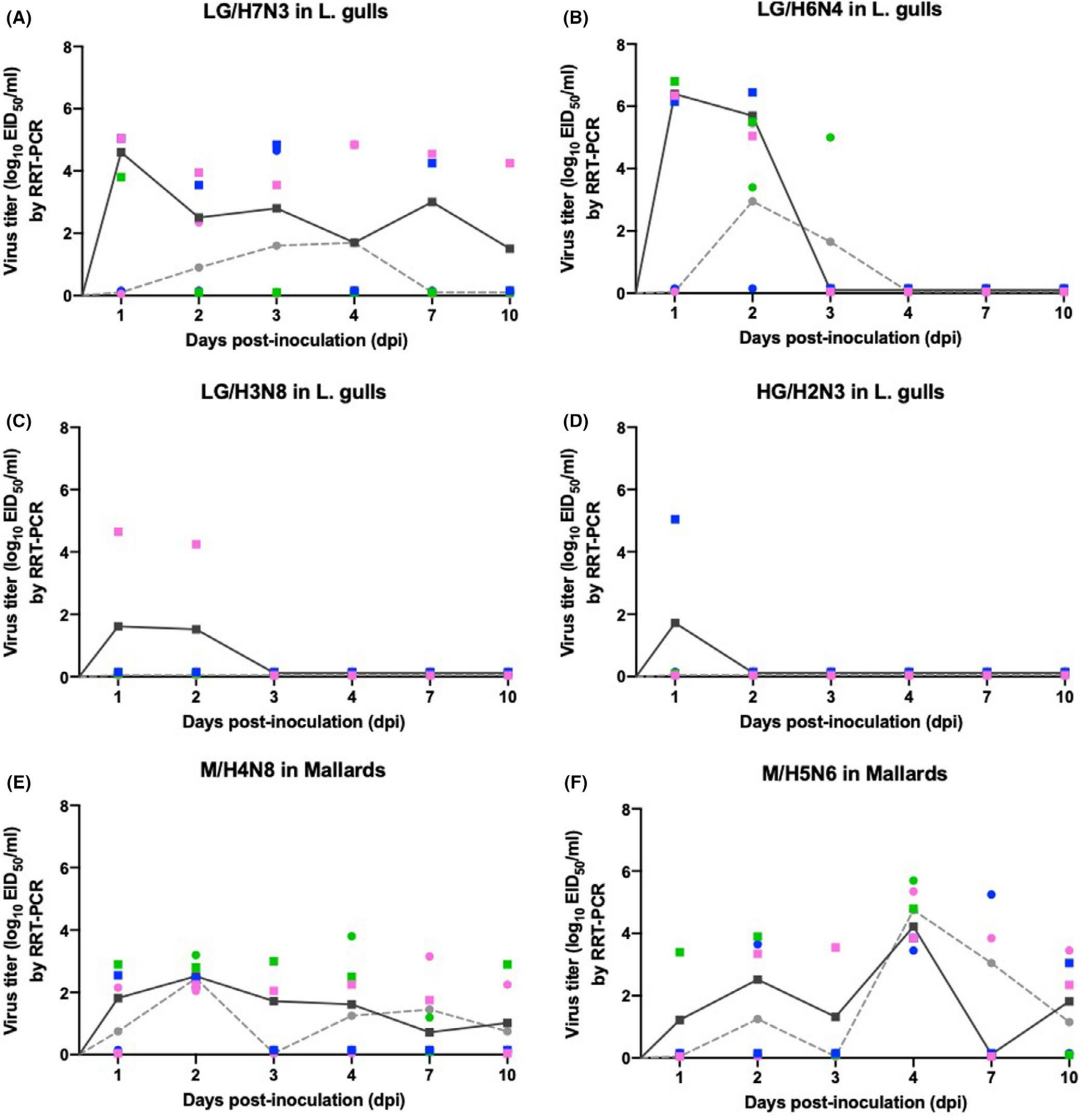
Evaluation of virus shedding in the oropharyngeal (OP) and cloacal (CL) swabs after different days post‐inoculation (dpi) of LPAIV in laughing gulls and mallards. Variation in viral shedding patterns observed in laughing gulls (A to D) and mallards (E and F) experimentally infected with different LPAI virus strains at dose ~10^6.0^ EID_50_/0.1 ml. Virus shedding titers, represented as log_10_ EID_50_/ml, were evaluated by RRT‐PCR on 1, 2, 3, 4, 7, and 10 dpi. Black (OP) and dashed gray (CL) lines indicated means per sampling day. Squares (OP) and dots (CL) indicate values for individual birds (n=3 birds per day). For each experiment, birds 1, 2, and 3 are shown in magenta, blue, and green color, respectively. Plotted data from each bird had a nudge of 0.05 in the Y direction for dataset visualization

Viral shedding patterns, including respiratory versus gastrointestinal tracts and duration, varied between the LPAIVs and individual birds (Figure 1A‐F). Gulls inoculated with LG/H7N3, and LG/H6N4 had the highest shedding titers, with mean OP titers reaching peaks of 4.5 and 6.3 log_10_ EID_50_/ml, respectively (Figure 1A‐B). Only LG/H7N3‐inoculated gulls shed to end of the 10 dpi while LG/H6N4‐inoculated gulls shed to 3 dpi. In contrast, laughing gulls infected with LG/H3N8 and HG/H2N3 (Figure 1C‐D) had low‐OP virus titers (highest mean virus titer detection of 1.5 and 1.7 log_10_ EID_50_/ml, respectively), and virus was not detected in CL swabs. Overall, in laughing gulls viral shedding was predominantly associated with OP swabs, with highest titers observed in the first two days of post‐inoculation. Virus was detected less frequently in CL swabs and titers were low (Figure 1). Mallards inoculated with M/H5N6 (Figure 1E), and M/H4N8 (Figure 1F) had viral shedding detected over the 10‐days study. In the first three dpi, the mean virus shedding was higher in the OP than in the CL samples for M/H5N6‐inoculated mallards (Figure 1E). This shedding pattern changed after 4 dpi with M/H5N6 virus excretion in the CL reaching peaks as 4.7 log_10_ EID_50_/ml (Figure 1E). Mallards inoculated with M/H4N8 had a constant mean shedding titer in the OP samples during the 10 dpi, and CL shedding oscillated between days and birds with titers similar between OP and CL samples or CL slightly lower than OP swabs (Figure 1F).

**FIGURE 2:**
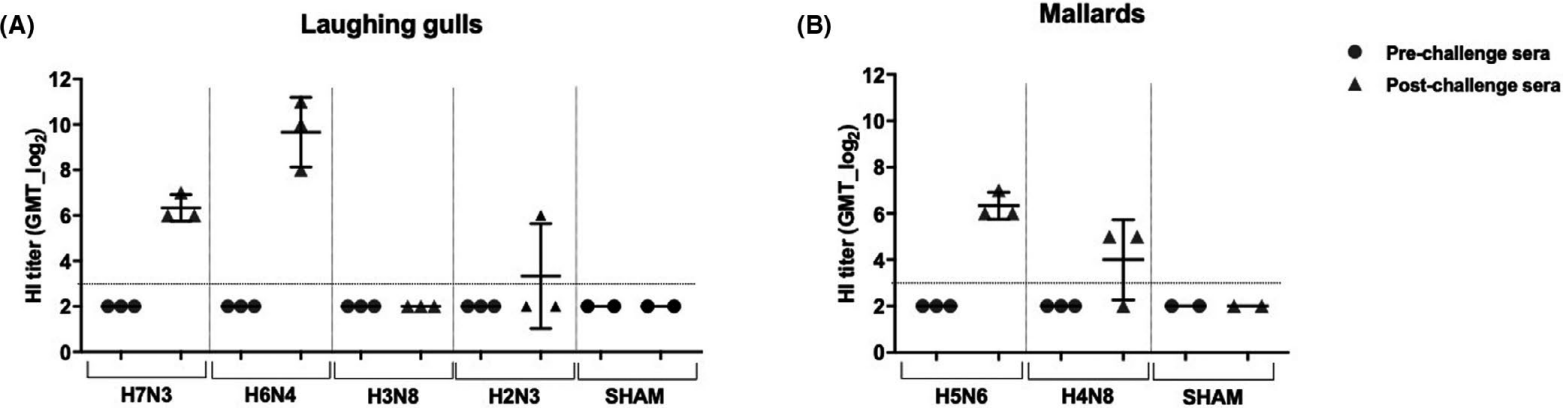
Scatter plot of HI titers in laughing gulls and mallards pre‐ and post‐challenge with different LPAI virus strains. The HI titers were analyzed using the challenge virus as antigen for the experimental infection in (A) laughing gulls and (B) mallards. Titers were expressed as geometric mean titers (GMT‐log_2_), and samples with titers below 3 log_2_ GMT were considered negative. Dotted horizontal lines indicate limit of detection

None of the laughing gulls or mallards had pre‐existing anti‐AIV antibodies prior to LPAIV inoculation (Table 2 and Figure 2). All gulls seroconverted following challenge with LG/H7N3 and LG/H6N4 and all mallards seroconverted following M/H5N6 challenge. Two of three mallards challenged with M/H4N8 seroconverted and only one laughing gull seroconverted after HG/H2N3 and LG/H3N8 challenge (Table 2).

## 4 DISCUSSION

Studies of LPAIV are crucial to provide an understanding of interactive association between LPAIVs, avian host, and the environment. This knowledge is needed to identify mechanisms related to LPAIV maintenance, subtype diversity, and evaluate the risk factors that contribute to LPAIV spread to new geographic regions or hosts, which includes other wildlife species, poultry, domestic animals, and humans. In this study, laughing gulls were inoculated with relevant LPAIV frequently detected in North American surveillance studies from gulls to determine the unknown infectivity, viral shedding patterns and pathogenicity. This data was contrasted with results from two mallard infections with H5N6 or H4N8 mallard‐origin viruses.

None of the LPAIV‐infected gulls or mallards in our experiments experienced morbidity or mortality (Figure 1 and Table 2) which was expected with LPAIV in these host species.^1^, ^8^ Gulls inoculated with LG/H7N3, LG/H6N4 and LG/H3N8, all become infected based on detection of their respective LPAIV in one or more OP or CL swabs, but only one laughing gull became infected when inoculated with HG/H2N3 obtained from a herring gull (*Larus argentatus*), a related gull species. The HI or bELISA antibody tests confirmed such infections in most inoculated gulls except for two gulls (Table 2), which despite having low‐virus replication and shedding titers, no anti‐AIV antibodies were detected by either method.

The most interesting outcome was subtle differences in virus shedding patterns and their implications on virus transmission and maintenance in gulls compared to mallards. In this study, and others, involving laughing, silver, and ringed‐billed gulls, predominate shedding of LPAIV from the oropharynx has been observed.^9^, ^10^, ^11^ With black‐headed gulls, peak prevalence of H13 and H16 viruses is associated with fledged birds during the breeding season and predominant OP shedding may represent an adaptation for efficient transmission during this period.^12^ Previous studies demonstrated LPAIVs infect and replicate in both respiratory and intestinal epithelial cells of mallards and domestic ducks (*Anas platyrhynchos domesticus*).^8^, ^13^, ^14^ In ducks, the high volume of feces containing high titers of LPAIV and the long duration of shedding would both contribute to contaminate aquatic habitats and facilitate fecal/oral transmission.^1^, ^8^ This predominant shedding pattern was reproduced in mallards in this study where ducks were experimental infected by intrachoanal inoculation, which simulates exposure during natural feeding behavior; both intrachoanal and direct gastrointestinal exposure have resulted in productive LPAIV infection in mallards.^13^ The observed differences in shedding by mallards and gulls inoculated by the same route, suggesting different mechanisms for transmission and maintenance of LPAIV in gulls and dabbling ducks.

It is possible that differences in shedding patterns between ducks and gulls may be related to the expression of α2,3‐linked sialic acid (SA) receptors in tissues.^15^, ^16^ Ducks show similar expression of SA receptors in the respiratory and intestinal tract, which may explain the equal respiratory and fecal shedding pattern of our study.^15^, ^16^ In vitro studies demonstrated that SA receptors’ stronger expression in respiratory tract of ring‐billed gulls and laughing gulls which was consistent with predominant respiratory shedding in our study.^15^, ^16^ However, other factors present in the host and the virus strain may be also involved in the differences in AIV prevalence, viral shedding, and the disease's outcome,^5^, ^15^, ^16^ which are beyond this study's scope.

The comparative study of different taxa of migratory aquatic birds, especially Chadriiformes and Anseriformes, offers a unique opportunity to understand how different LPAIV subtypes evolved and are maintained in diverse avian ecosystems. Our data demonstrated different patterns of viral shedding associated with relevant LPAIV subtypes in laughing gulls as compared to current understanding in mallards. Further studies are needed to understand the pathophysiological and ecological mechanisms of AIVs transmission cycles in gulls to better understand cross‐species transmission and environmental maintenance.

## CONFLICT OF INTEREST

Authors declare no conflict of interest.

## AUTHOR CONTRIBUTIONS

**Miria F. Criado:** Conceptualization (equal); Data curation (equal); Formal analysis (equal); Investigation (equal); Methodology (equal); Validation (equal); Visualization (equal); Writing‐original draft (lead); Writing–review & editing (supporting). **Kira A. Moresco:** Conceptualization (equal); Data curation (equal); Formal analysis (equal); Investigation (equal); Methodology (equal); Validation (equal); Visualization (equal); Writing‐original draft (supporting); Writing–eview & editing (supporting). **David E. Stallknecht:** Conceptualization (supporting); Data curation (supporting); Formal analysis (supporting); Methodology (equal); Validation (equal); Writing‐review & editing (supporting). **David E. Swayne:** Conceptualization (equal); Data curation (equal); Formal analysis (equal); Funding acquisition (lead); Investigation (equal); Methodology (equal); Project administration (lead); Resources (lead); Supervision (lead); Writing‐review & editing (supporting).

## Data Availability

The data that support the findings of this study are provided in the figures and tables of the article. Additional information is available from the corresponding author upon reasonable request.
